# Using RE-AIM and CFIR to develop a subsidized doula program for at-risk underserved pregnant mothers

**DOI:** 10.3389/fpubh.2026.1860925

**Published:** 2026-08-21

**Authors:** Leticia Aguerre Gatus, Kendra Folh, Audrey Kobina, Tajudaullah Bhaloo

**Affiliations:** 1 Community Health Network, Institute for Health Access and Engagement, Memorial Hermann Health System, Houston, TX, United States; 2 Women and Children’s Service Line, Memorial Hermann Health System, Houston, TX, United States; 3 Nursing Excellence and Clinical Excellence, Memorial Hermann Cypress Hospital, Cypress, TX, United States; 4 Institute for Nursing Excellence, Memorial Hermann Health System, Houston, TX, United States

**Keywords:** doula, health disparities, implementation science, interdisciplinary team, maternal healthcare

## Abstract

The paper describes how a large healthcare organization used RE-AIM and CFIR frameworks to systematically guide the program planning for an evidence-based doula program. Despite the known benefits of doulas, disparities persist, specifically among vulnerable individuals. The program embraced a holistic approach that coupled screening and nurse navigation with doula care to at-risk pregnant individuals. Identified barriers and facilitators influenced program design to improve reach and effectiveness. The paper presents how RE-AIM and CFIR can be used to design a comprehensive doula program, aimed to address an ongoing public health crisis.

## Introduction

Over the last two decades, while maternal mortality has declined in other high-income countries, it has become a public health crisis in the US (1). Black (non-Hispanic) women in the US have 2–4 times higher risk of death after childbirth compared to White women (1–3). Tragically, almost 80% of these deaths were considered preventable (4, 5). Even when controlling for education and co-morbid conditions, Black (non-Hispanic) women are up to 3.3 times more likely to die from complications associated with their delivery or pregnancy, compared to White women without higher education (1, 6).

Even more concerning is that for every maternal death, approximately 100 women experience severe maternal morbidity (SMM), defined by the American College of Obstetricians and Gynecologists as “unintended outcomes of the process of labor and delivery, resulting in significant short-term or long-term consequences to a woman’s health,” (33). Several factors contribute to the higher rates of both maternal morbidity and mortality, including personal health related contributors (e.g., clinical risk factors, maternal age, pre-existing health issues and lifestyle/ behavioral factors), organizational considerations (e.g., access to health care, provider biases) and system and policy factors (e.g., structural racism, or system impediments such as accessing social protection programs or resources) (5). Black individuals are also more likely to face a variety of social determinants of health challenges including, but not limited to: food insecurity; insufficient access to pre-natal care; and possess lower levels of education (7, 8).

### Maternal morbidity and mortality in Texas

This national public health crisis is particularly problematic in Texas, the second largest state, which makes up approximately 10% of the nations’ births (9). Sadly, Texas’ maternal mortality increased by approximately 56% in 2022, compared to an 11% increase across the US in the same time frame (10). As of 2020, non-Hispanic, Black women in Texas experienced SMM at almost double the rate of non-Hispanic, White women (i.e., 117.3 Black women experience SMM per 10,000 delivery hospitalizations compared to 56.3 White-non-Hispanic women) (11). Persistent health disparities and high rates of uninsured individuals partially explain why Texas is also a leader in pre-term births, babies born with low birth weight and has the lowest rates of women receiving prenatal care in their first trimester compared to national averages (e.g., 67.7% of Texas mothers in 2020 vs. 76.1% nationally) (5, 12). Forty-six and half percent (46.5%) of Texas counties (vs. 32.6% of counties in other US states) are ‘maternity care deserts’, defined as no access to a birthing facility or maternity care professional, which perpetuates disparities at the city and county level, contributing to poor health outcomes for pregnant women and their children (13, 14). Texas’ high rates of maternal mortality and morbidity and its large ethnic, racial and linguistic diversity were important factors that prompted the Texas Department of State Health Services to develop recommendations, which included promoting doula care and home visiting programs to better support the health needs of mothers and infants (9, 15).

### Rationale for doula care

Doulas not only offer emotional, physical and culturally sensitive support, but they also educate clients on pertinent health literacy topics, advocate for their clients and promote positive health behaviors during the intrapartum period (3, 16, 17). Research confirms that doula care is associated with improved maternal health outcomes for high-risk/at-risk mothers. For example, studies using national or multi-state Medicaid claims data report that doula care was associated with 47% lower risk of cesarean delivery, 29% lower risk of low birth weight, 22% reduction in preterm births, 46% increases likelihood of accessing postpartum primary care and 57.5% lower odds of presenting with postpartum depression or postpartum anxiety (3, 18). Studies associated doula care with reduced need for analgesia and oxytocin during labor, decreased likelihood of low 5-min APGAR scores and higher rates of exclusive breastfeeding (19, 20). Additional patient-related benefits of doula care documented in the literature include improved patient self-esteem, increased patient satisfaction, both of which contributed to improved birthing experiences (20). The desire to reduce SMM, lower cesarean section rates, improve birth equity and patient experience among underserved women in the Greater Houston region prompted a non-profit integrated health care system to conceptualize and implement a subsidized doula program to promote maternal health within the community.

## Using RE-AIM and CFIR frameworks

Dissemination and implementation frameworks, such as *RE-AIM* (Reach, Effectiveness, Adoption, Implementation and Maintenance) and *CFIR* (Consolidated Framework for Implementation Research), have been widely used individually, as well as together, to inform planning, implementation, program modification and even evaluation of health programs (21). The five domains of RE-AIM are: Reach (i.e., intended beneficiaries), Effectiveness (anticipated benefits our intended outcomes), Adoption (where the intervention is being implemented and context), Implementation (how the intervention is delivered, monitored for fidelity and appropriate for the context) and Maintenance (how and when the program will be integrated into operations and sustained) (21, 22). Commonly used in public health, RE-AIM has also been used in community and clinical practice settings because of its practical application to program design and focus on external validity (21). CFIR, a widely used implementation science framework, provides a systematic way to assess potential barriers and facilitators, examine organizational readiness, guide planning and/or implementation through five domains and 39 constructs associated with effective implementation (23, 24, 32). The five CFIR domains include: intervention characteristics (of the innovation), inner setting (factors internal to the organization), outer setting (factors outside of the organization), characteristics of individuals (e.g., beneficiaries) and processes (strategies associated with implementation).

These frameworks, when used together, highlighted diverse interconnected factors and prompted program developers (or evaluators) to consider multi-level factors—all of which influenced outcomes, success and were associated with improved knowledge translation of evidence (21). Studies, which have used these frameworks together, did so retrospectively, i.e., study implementation planning, examine factors that influenced implementation, or evaluate implementation (21, 25–27). Researchers have used the RE-AIM framework to define program outcomes and then applied the CFIR framework to review successes and barriers that influenced program outcomes (26, 27). The combination of these two frameworks have enabled researchers to: identify system-level factors that impacted implementation; understand the experiences of multiple settings implementing the same evidence-based intervention; track ongoing implementation; and/or develop important lessons for sustainability and/or replication, especially for programs serving vulnerable populations. Equipped with evidence of doula programs which improved maternal health, organizational leadership leveraged these two frameworks to inform program design and planning; this paper adds to the literature demonstrating how these frameworks can be used prospectively.

### Program design using RE-AIM

State, regional and organizational data formed the body of evidence, which influenced overarching goals to reduce maternal mortality and morbidity by connecting low-income and at-risk women to a doula, trained to offer emotional and health literacy support. The program’s goals were to: (1) improve maternal health for eligible women (defined as reduced SMM, unplanned Cesarean sections, and fewer maternal readmissions among program participants and reduced neonatal injuries); and (2) improve patient experience and connection to resources and health information (e.g., by having doulas and nurses provide emotional, educational, physical and navigation support throughout pregnancy, childbirth, and the postpartum period).

An interdisciplinary Program Steering Committee, with representation from diverse leaders from across the integrated health care system, used the RE-AIM framework to guide the program design and planning outlining the “*who,” “what,” “how”* and “*where”* of the program. The first component of the framework, *Reach*, prompted the leadership to elaborate on the intended “underserved” target population, defined as: women belonging to racial or ethnic minority groups; women on Medicaid or who report an annual income under $75,000; women living in specific zip codes with a known history of high maternal morbidity and/or mortality; first time mothers; women previously identified by the health system to have more than 2 social drivers of health (e.g., food insecurity, transportation challenges); pregnant individuals under age 18 or over age 35; and pregnant women with co-morbid conditions (e.g., diabetes, obesity, hypertension, previous Cesarean section, a prior traumatic birth experience). In contrast to most studies which have exclusively focused on Medicaid beneficiaries (3), the organization used an expanded definition of at-risk based on community need.

*Effectiveness,* guided leadership to reflect on program intervention components that would be important to achieve intended outcomes.

#### Holistic, patient-centered approach

A holistic, patient-centered program needed to include the following components: screening for non-medical drivers of health; navigation to community programs and services; individualized patient support; patient education for improved health literacy; care coordination; and advocacy for program beneficiaries (patients and families). Recognizing that eligible program enrollees could have a multitude of unmet needs, the program was designed to be complementary to other medical and pre-natal care services available to program participants.

Doula services were designed to begin during pregnancy (i.e., any time after 10 weeks) and extend up to 1 year post-delivery, i.e., the intrapartum period. Perinatal nurse navigators received referrals, confirmed eligibility and conducted social determinants of health screening, before partnering the enrolled patient with a doula.

Doulas had at least one in-person visit during pregnancy, which enabled them to develop a relationship with the patient, offer emotional support, promote prenatal care, and support individual concerns and questions during the pregnancy. In-person visits were supplemented with phone conversations and text messaging; doulas were accessible to patients even during non-traditional hours. Doulas connected enrollees to evidence-based information and community resources, worked with patients to create a birth plan and even helped them develop questions for their doctors’ visits. Doulas offered patient-centric services, which was instrumental in creating a trusting, supportive relationship, focusing their time on issues important to each patient. Doulas were present for labor and delivery, supported patients and their families during the birthing process, helped explain deviations from the birth plan and/or were instrumental in incorporating patients’ wishes, in spite of unexpected deviations from the birth plan.

The holistic approach was especially important postpartum, when doulas supported patients with recovery and breastfeeding. After delivery (doula) home visits were dedicated not only to the mothers’ physical and mental health, but also the infant’s care and well-being (e.g., breast-feeding support, general infant care). During these post-birth interactions, doulas often uncovered unexpected patient symptoms or needs; perinatal nurse navigators were an important partner for doulas connecting patients to mental health support, medication resources or specialty care.

**Figure 1:**
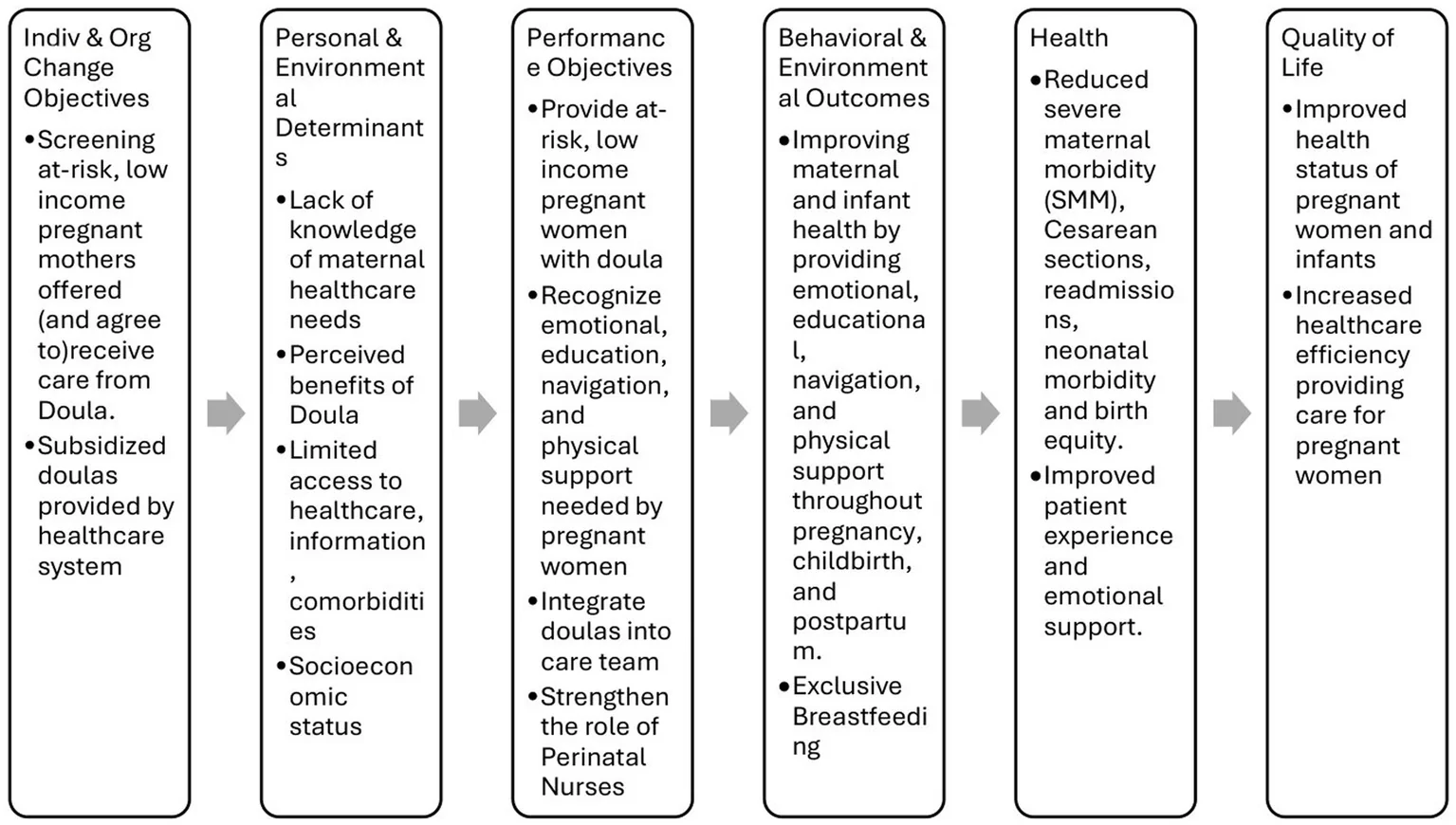
Doula program intervention logic model.

The program logic model, developed by select program leaders and the authors, Figure 1 demonstrates how important intervention components connect to desired outcomes, defined as reduction in severe maternal morbidity, (unplanned) Cesarean sections, readmissions and neonatal injuries.

*Adoption*, defined as factors and characteristics of the intervention setting, included how those involved in the intervention received it. As a multi-site, integrated health care system, program leadership opted for phased-in implementation, beginning with only three (out of 14) hospital sites, selected for their familiarity with doulas (i.e., doula -friendly providers and nurses), and for geographic proximity to where doulas were already being utilized. These initial hospital sites were identified as early-adopters and had strong relationships with the Women and Children’s Service Line, the program structure responsible for designing and leading the program creation and implementation. The program expanded to the remaining 11 hospitals sites over the course of a 1 year time period. The organization’s significant volumes of deliveries each year and expertise among staff and physicians, coupled with the desire to render quality women’s care supported implementation efforts. Existing relationships with community organizations, such as the March of Dimes, which had already developed a reputation for its doula training, were important factors that promoted adoption.

As for *Implementation,* the interdisciplinary leadership structure included representation from diverse corporate and clinical areas, as well as leaders from participating hospitals. Frequent and consistent communications and presentations at system-wide leadership councils kept pertinent stakeholders informed as the program was conceptualized. Ensuring nurses, leaders, other staff and physicians were informed of program elements was an enormous and complex task in a multi-site organization; program leadership leveraged multiple communication vehicles, including, but not limited to: oral communication in multi-site forums, email communication for pertinent staff, discussions and updates during unit-specific staff and clinical meetings, flyers available on nursing units, information on the organization’s intranet and external website, and direct physician office communications. The leadership successfully secured grant funding for the initial year-long pilot, in order to offer subsidized doula care; a critical part of the pilot program was that eligible mothers did not pay for doula care. Grant funding ensured that doulas were remunerated per industry rates.

In collaboration with the March of Dimes, six community doulas, who were certified and had at least 1 year of experience as a doula, were recruited; these were mentored by another seasoned doula, who also managed workload and patient assignments. Human resources, legal consultants and nursing leaders collaborated and created a standardized doula role description and scope of practice. Doulas participated in new hire onboarding and patient experience training provided to all healthcare system employees, in order to orient them to organizational policies as well as service and care standards. These were instrumental in integrating doulas into hospital operations and culture.

For *Maintenance,* program leadership from the Women’s Service Line continued communication and education efforts to ensure all hospital sites were engaged and informed as the program expanded to new hospital sites by partnering with Marketing team and targeted physician offices. Monthly meetings between program leadership and the doulas created a forum to discuss operational issues. Recruitment and enrollment were refined to ensure continuous enrollment of potential eligible patients. Lastly program leadership have proactively found additional funding to sustain the program beyond the initial one-year pilot. The RE-AIM framework prompted the leadership to focus on multiple domains as maternal morbidity and mortality is a complex public health issue and ensure alignment between the target group and subsequent strategies on how to serve the target group (See Table 1).

**Table 1:** RE-AIM framework and program strategies.

RE-AIM framework	Strategies
Reach	Target population were at-risk individuals defined as: low-income; at-risk pregnant women belonging to racial or ethnic minority groups; women on Medicaid or who report an annual income under $75,000; women living in specific zip codes with a known history of high maternal morbidity and/or mortality; first time mothers; women previously identified by the health system to have more than 2 social drivers of health (e.g., food insecurity, transportation challenges); pregnant individuals under age 18 or over age 35; and pregnant women with co-morbid conditions (e.g., diabetes, obesity, hypertension, previous Cesarean section, a prior traumatic birth experience).
Effectiveness	Holistic support combining doula care with nurse navigation through intrapartum period (prenatal, delivery and postpartum); designed to reduce severe maternal morbidity (SMM), unplanned Cesarean sections, readmissions, neonatal injuries and promote exclusive breast feeding. Funding subsidized doula care. Doulas offered personalized support, health literacy, and advocacy.
Adoption	Alignment with organizational vision and mission; leverage existing expertise in maternal health. Phased-in implementation, launching the program at 3 hospitals, and expanding to the remaining 11 hospitals. Comprehensive training and collaboration: Doulas received the onboarding and additional training to function within the integrated health care system.
Implementation	Standardized role description and scope of practice. Training and mentorship for doulas and how they supported at-risk program enrollees. All doulas were recruited as hospital employees & experienced consistent onboarding training and employee orientation (“culture day”).
Maintenance/sustainment	Program leadership have been proactive in finding additional funding to sustain the pilot program beyond the initial one-year timeframe. Recruitment and enrollment processes have been refined with the support of multiple departments to ensure a continuous influx of at-risk potential enrollees. Integration into the healthcare system: The Women’s Service Line partnered with Marketing and targeted physician offices for on-going to educate and recruit women.

### Program design using CFIR

For the purposes of this work, the authors focused on three components of the CFIR framework: the Outer Setting (external factors that influence implementation), Inner Setting (internal organizational factors such as resources, readiness for change and organizational culture) and Individuals (attributes of the target population that influenced implementation). CFIR provided a systematic way to guide implementation planning, assessing for potential barriers and facilitators of implementation (23). See Table 2 for CFIR domains and corresponding programmatic strategies used in designing the Doula Program.

**Table 2:** CFIR domains and strategies.

CFIR framework domains	Strategies
Outer setting	Recognition that maternal mortality and morbidity remained high March of Dimes and other community partners had expertise, resources and reach within targeted population Increasing acceptance of doulas and alternate professionals to support child birth External grant funding to subsidize doula time.
Inner setting	Work/Leadership Infrastructure (organizing work among Clinical Care Team + Doula) Initial hiring of 6 doulas Informal and formal communications Relational Connections Enhancing patient experience Prior doula/nurse training at early adoption sites.
Individuals	Innovation Recipients (pregnant women) Innovation Deliverers (Doulas) Implementation Team Members (Care Team) High-Level Leaders (Women Service Line and physicians).

As part of the *Outer Setting*, the program steering committee leveraged data on SMM, non-medical drivers of health and the organization’s clinical data, which supported a holistic approach that extended beyond only doula care. Although maternal morbidity and mortality is a complex public health issue, several studies have validated the benefits associated with doula care, providing evidence of how doulas overcome individual barriers and build trusting relationships (28, 29). Key community partners, such as the March of Dimes, were instrumental in doula recruitment and marketing the program to the intended target population. Recruited doulas understood the social, behavioral and health needs of the target population, had been working in the community for at least 1 year, wanted stable/predictable income and were individuals who wanted to be associated with a hospital system. Doulas leveraged their relationships with churches and other non-profits to support marketing and patient recruitment.

*Inner Setting* factors included internal leadership structures that guided the implementation, formal and informal communication and workflow and cultural norms. Program leadership used the Adams Influence Model (30) as part of change management strategies. As an integrated health care system with a long history of caring for pregnant individuals and the largest volumes of births in the region, leaders came together around a shared value of enhancing patient experience. Leaders recognized that offering intrapartum doula care was a significant and complementary approach to other strategies in-place, which included increasing access to prenatal care, promoting provider bias training and critically reviewing patient feedback from post-discharge surveys. One of the most significant anticipated challenges was how to appropriately integrate doulas into hospital birthing teams, especially given the large number of nursing staff. Program leadership embraced multiple communication strategies, which included targeted email communication about the program to nurses and other stakeholders, system-wide announcements on the organization’s intranet, and marketing collateral on the organization’s website. Existing nurse navigators, who leveraged their personal relationships with nursing staff and clinical leaders, helped with in-person introductions between labor and delivery and postpartum nursing staff and doulas (allowing them to meet each other). Flyers, which included pictures of the doulas and an excerpt about their background, helped familiarize the nursing staff with these new care team members. Doulas were provided a standard script, which they used to introduce themselves to nurses when then came into the hospital at the time of the delivery. Leadership conversations identified challenges, such as individual hospital policies on access to operating rooms; while some hospitals permitted doulas to accompany pregnant individuals into the operating room, others did not. Given the autonomy of each participating hospital, the program had to respect the policies and practices of each site.

The *Individuals* domain helped identify key stakeholder groups, elaborating on how each of these groups could be impacted by the intervention and/or how they could uniquely support the implementation. For example, for program enrollees (pregnant women), CFIR helped highlight possible barriers such as how to reach and identify intended program enrollees, possible transportation barriers and limitations relating to access to resources and information; these prompted the program to create multiple communication vehicles (including using partners to market the program in the community), leveraging the organization’s large patient base (through social media platforms) and prompting doulas to offer in-person and online support to make it easier to cater to program enrollee needs. Especially relevant in the postpartum period, doulas gave program participants the option to continue support sessions virtually to accommodate individual circumstances. Hospitals selected for early adoption either had staff that had experience working with doulas or had offered doula training to their nurses to enhance the understanding of the role and scope of doula care. A single electronic medical record, with embedded screening tools, supported screening for social determinant of health issues, understanding personal and social histories and navigation. Perinatal nurse navigators were identified as another stakeholder group; their experience and relationships with nursing staff was identified as a resource as nurses respected and trusted their judgment. Perinatal nurse navigators were advocates for the program and actively helped explain how doulas could improve patient experience among the target group. Physicians, i.e., hospitalists, obstetricians affiliated with the organization and anesthesiologists were another important stakeholder group; hospital and program leaders made dedicated efforts to communicate with these groups through electronic and in-person communication. It was challenging to inform affiliated physicians, and the program relied on just-in-time and ongoing communication from hospital leaders to help address questions and concerns. These conversations were helpful in permitting doulas to remain with patients who needed a surgical intervention.

### Doula program logic of change

The doula program logic model describes how specific change determinants, changes in behavior, and environmental factors connect to intended health and quality of life outcomes (Figure 1). At-risk mothers, screened for eligibility, were partnered with a subsidized doula. The at-risk mothers’ determinants included factors such as lack of maternal healthcare knowledge, socioeconomic status, and comorbidities. The performance objectives included multiple strategies such as integrating the doulas into the clinical care team, responding to mothers’ educational and emotional needs, and offering navigation. The intended program outcomes included improved maternal and infant health, improved patient experience as well as appropriate utilization of health care services. The impact on health included reductions in severe maternal morbidity among enrollees, reduced unplanned cesarean sections, and reduced neonatal morbidity.

## Discussion and implications

RE-AIM and CFIR frameworks guided the planning and early implementation of a subsidized doula program designed to improve the health of underserved women seeking care from a large multi-site hospital system. These frameworks gave structure to our planning efforts and helped identify factors that influenced eligibility criteria (reach), defined core program interventions, highlight aspects that could facilitate adoption and influence sustainability. The program design was built on internal expertise and leveraged external partnerships and resources.

Studies which have used both the RE-AIM and CFIR frameworks, have used them to evaluate implementation efforts. Although CFIR has been applied in diverse healthcare settings, it has not been applied to maternal health programs. To our knowledge, only one other maternal health study leveraged CFIR (31). Our work serves as an example of how to use both frameworks to inform program design and program planning to address a complex public health issue.

Studies that used both RE-AIM and CFIR together in post-implementation evaluations demonstrated how programs targeting vulnerable populations often have limitations in program reach or other access barriers (21, 25). These studies emphasized the importance of non-medical drivers of health, which can improve program success. The holistic approach, which coupled screening and navigation with doula care, is one important way this program differed from other published doula programs. The partnership between nurse navigators and doulas supported care coordination and connected patients with resources before issues escalated into urgent needs.

Offering subsidized care and anticipating (patient) transportation barriers were two of the most impactful adaptations made (at the individual level) to enhance access. These two aspects would have otherwise impeded access to doula services for underserved patients. Additional facilitators included steadfast leadership support, standardized doula role descriptions, and consistent onboarding and training, which facilitated integration within healthcare teams. These strategies also supported the long-term sustainability of the program.

### Practice and care delivery implications

The initial plan to align doulas with particular hospital sites (to promote relationship building with hospital teams) proved impractical in the early phases of the program, as doulas were partnered with patients as patients enrolled across multiple sites simultaneously. However, this could be a strategy as the program matures and evolves over time.

In our pilot program, doulas were English speaking, however, given the significant Spanish-speaking population in our community, future iterations of the program need to introduce Spanish-speaking doulas, in order to offer linguistically sensitive care and reach underserved individuals, who may not be currently well served in the community.

## Limitations

The limitations of the Doula Program were that it was primarily based in Harris County with only six doulas to cover multiple hospitals. The program was implemented intentionally at three hospitals, where staff and providers were more familiar with doulas and their role, however the number of doulas remained constant as the number of sites increased. Keeping stakeholders informed as the program expanded to additional hospitals proved to be an enormous undertaking. As the health care system serves multiple counties, the program will need to be flexible and adapt to each hospital’s culture and unique patient community. While additional funding has been secured to continue the program, funding is a critical issue that has yet to be resolved to sustain the program. While the current funding subsidized the cost of doulas’ time, initial pilot findings may not be generalizable to the health systems’ broader patient population.

## Future directions

More work is needed to fully evaluate the program, demonstrate its value to health systems and funders, and validate that the investment in doula care results in enhanced patient experience and improved health outcomes for the mother and child.

Since doula care can be cost prohibitive for pregnant women, future sustainability strategies include aligning financing structures with workforce models for long-term sustainability and scalability. For example, one approach involves incorporating doula services into bundled payments offered by local employers. This approach positions doula support as a standard component of maternity care rather than an ancillary service, particularly in employer-sponsored populations seeking improved outcomes and reduced total cost of care. Other possibilities include exploring the integration of doula-informed training and competencies into existing Patient Care Technician (PCT) roles within obstetric units. This strategy leverages the existing workforce to extend culturally responsive, continuous labor support without requiring a fully separate staffing model. By cross-training PCTs in core doula skills—such as patient advocacy, non-clinical labor support, and emotional care—the model promotes scalability through role optimization and workforce flexibility while maintaining continuity within hospital-based care teams. Last, but not least, the leadership are considering the incorporation of doulas as Community Health Workers (CHWs) within the organization’s community benefit infrastructure represents another sustainable design pathway. This model bridges clinical care and community-based support, allowing doulas to operate across care settings and address social determinants of health that significantly impact maternal outcomes. Positioning doulas within CHW frameworks also opens access to emerging reimbursement mechanisms at the state and payer level, particularly those tied to preventive services, health equity initiatives, and Medicaid-funded community-based care. The need to align maternal health outcomes with cost and quality incentives and value-based purchasing arrangements, are a plausible and viable pathway for ongoing reimbursement beyond grant funding.

## Data Availability

The original contributions presented in the study are included in the article/supplementary material, further inquiries can be directed to the corresponding author.
